# Fetal hypoxia and apoptosis following maternal porcine reproductive and respiratory syndrome virus (PRRSV) infection

**DOI:** 10.1186/s12917-021-02883-0

**Published:** 2021-05-01

**Authors:** Carolina M. Malgarin, Fiona Moser, J. Alex Pasternak, Glenn Hamonic, Susan E. Detmer, Daniel J. MacPhee, John C. S. Harding

**Affiliations:** 1 Western College of Veterinary Medicine, Saskatoon, 52 Campus Dr, Saskatoon, Saskatchewan S7N 5B4 Canada; 2 Department of Animal Science, Purdue University, West Lafayette, USA

**Keywords:** Swine, Fetus, Apoptosis, Hypoxia, TUNEL, PRRS, Gene expression

## Abstract

**Background:**

Mechanisms of fetal death following maternal PRRSV2 infection remain uncharacterized, although hypoxia from umbilical cord lesions and/or placental detachment due to apoptosis are hypothesized. We performed two experiments examining hypoxia and apoptosis in PRRSV-infected and non-infected, third-trimester fetuses to elucidate possible associations with fetal death. Fetuses were selected based on four phenotypic infection groups: fetuses from non-challenged control gilts (CTRL); low viral load fetuses (LVL; Exp 1) or uninfected fetuses (UNINF; Exp 2) from inoculated gilts; viable high viral load fetuses (HVL-VIA); and HVL meconium-stained fetuses (HVL-MEC).

**Results:**

In experiment 1, paraffin embedded fetal tissues collected 21 days post maternal infection (DPI) were examined for DNA fragmentation associated with apoptosis. Positively stained foci were larger and more numerous (*P* < 0.05) in heart, liver, and thymus of HVL-VIA and HVL-MEC compared to CTRL and LVL fetuses. In experiment 2, group differences in gene expression within the hypoxia (HIF1a, IDO1, VEGFa, LDHA, NOS2, NOX1) and apoptosis (CASP3, CASP7, CASP8, CASP9, RIPK1, RIPK3) pathways were assessed by RT-qPCR in fetal tissues collected at 12 DPI. High viral load fetuses showed differential expression relative to the CTRL and UNINF (*P* < 0.05 for all). Brain tissue from HVL-VIA and HVL-MEC fetuses presented increased expression of CASP7, CASP8, RIPK3, HIF1a and IDO1. Fetal heart showed increased expression of CASP8, HIF1a, IDO and NOX1 and a decrease in NOS2 expression in infected groups. CASP7, CASP9, RIPK1 and RIPK3 were only increased in the heart of HVL-VIA while VEGFa was only increased for HVL-MEC fetuses. Thymus from HVL-MEC had decreased expression of CASP9 and there was increased IDO1 in all infected fetuses.

**Conclusions:**

There is strong evidence of apoptosis occurring in the heart, liver and thymus of highly viral load fetuses at 21 DPI. Furthermore, there was clear upregulation of apoptotic genes in the heart of high viral load infected fetuses and less prominent upregulation in the brain of PRRSV-infected fetuses, whereas thymus appears to be spared at 12 DPI. There was no strong evidence of hypoxia at 12 DPI in brain and thymus but some indication of hypoxia occurring in fetal heart.

**Supplementary Information:**

The online version contains supplementary material available at 10.1186/s12917-021-02883-0.

## Background

Porcine reproductive and respiratory syndrome (PRRS) remains the most impactful viral pig disease in North America due to the heavy financial burden it has caused the pork industry [1]. Responsible for 45% of the economic losses, the reproductive form of the disease following PRRSV infection of sows during late gestation is characterized by abortions, fetal death, weak-born fetuses, and high pre-weaning mortality [2]. The mechanisms of fetal disease are not entirely understood and few studies have explored this topic, in part due to the lack of obvious fetal lesions [3, 4]. Following maternal infection, PRRSV rapidly infects the endometrium and crosses the epitheliochorial placenta to the fetus [5, 6].

We have previously reported that fetal serum is infected by 5 days after maternal inoculation (DPI) and fetal thymus by 8 DPI when fetal compromise first appeared [6]. Although gross and histopathologic lesions of infected fetuses have been characterized previously [3, 4, 7, 8], few studies have investigated possible mechanisms leading to fetal death after maternal PRRSV infection.

Due to the low frequency and inconsistency of fetal lesions, many studies have explored the maternal-fetal interface (MFI) for pathophysiological factors affecting fetal viability. Inflammatory lesions, placental detachment from endometrium, and apoptosis have been explored in both endometrium and placenta; however, none have proven directly responsible for fetal death [7–13]. Due to the infrequent but consistent finding of peri-vascular umbilical lesions in dead fetuses, hypoxia has also been proposed as a leading cause of fetal losses, due to the blood flow disruption [7, 14]. This is supported by the observation that the odds of meconium-staining of fetal skin [8], an indication of fetal stress and early clinical sign of fetal compromise, is associated with the presence of umbilical cord and fetal lesions [8].

Apoptosis is a normal physiological process responsible for programmed cell death in a selective manner, which when deregulated can lead to pathology and death [15]. Frequently used to investigate apoptosis, the terminal deoxynucleotidyl transferase (TdT) dUTP nick end labeling (TUNEL) assay detects DNA fragmentation due to apoptosis. TUNEL has been previously applied in PRRSV studies to assess severity of apoptosis in infected tissues and to associate the severity of apoptosis to PRRSV viral load in placental tissue from infected pregnant gilts at 21 DPI [13]. In addition, this methodology has been used to demonstrate that PRRSV cell infection not only results in apoptosis of the infected cells, but also induces apoptosis of surrounding non-infected cells at 10 DPI [9].

Although the pathophysiologic events occurring in the endometrium and placenta following maternal infection may contribute to fetal compromise, fetal death may also involve events compartmentalized to the fetal side. The absence of fetal lesions in many compromised and dead fetuses [8] raises questions about events occurring at a cellular and subcellular level in fetal tissues. RNA-Seq has been utilized and shown that the fetal thymus responded to infection by mounting an innate immune response, followed by an inflammatory response [16]. In a targeted gene expression study [17], IFNB, IFNG, CCL2, CCL5, CXCL10 and IL10, were upregulated in fetal tissues of high viral load fetuses at 21 DPI, although only CCL5 was elevated in more than one tissue from high viral load meconium-stained fetuses compared to high viral load viable fetuses, indicating that fetal immune response is not the main cause of fetal death. Nonetheless, a suppression of the cell cycle coupled with an increase in cardiac stress were found in the hearts of high viral load fetuses at 21 DPI [18]. More recently, differential expression analysis of some 283 immune related genes using the NanoString platform on placental and thymic tissues from fetuses at different stages of infection demonstrated response in either tissues was only initiated following infection of the fetus per se [19].

With these indications that fetal demise might be influenced by events occurring not only on the maternal side, but also in the fetal compartment, we aimed to determine if there was evidence of apoptosis and hypoxia in fetal tissues and if it was more pronounced in compromised (meconium-stained) fetuses. We designed two studies, firstly to assess apoptosis in fetal tissues collected between PRRSV-infected and non-infected fetuses at 21 days post-infection (DPI); and secondly, to determine differences in gene expression related to apoptosis and hypoxia in PRRSV-infected and non-infected fetuses collected at 12 DPI. Both experiments used the best available tissues archived from PRRSV pregnant gilt challenge studies conducted at the University of Saskatchewan, Canada.

## Results

### Experiment 1: differences in apoptosis in fetal tissues at 21 DPI

**Fig. 1:**
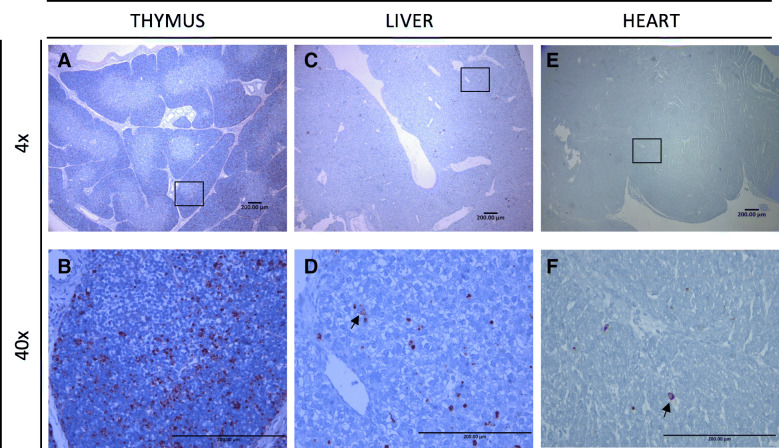
TUNEL staining in three fetal tissues of PRRSV-infected fetuses. **A** Diffuse areas of apoptosis on fetal thymus; **B** Higher magnification fetal thymus to display apoptosis (arrow) in thymocytes; **C** Areas with multiple apoptosis staining on fetal liver; **D** Higher magnification fetal liver to display apoptosis (arrow) in hepatocytes; **E** Focal spots of apoptosis in the fetal heart (arrow); **F** Higher magnification fetal heart to display apoptosis (arrow) in myocytes

In total, 100 fetuses were analyzed for apoptotic foci in LVR, THY and HRT. Only one fetus (HVL) had no TUNEL positive staining in THY, although its LVR had many TUNEL positive TUNEL foci. Only 8 fetuses had TUNEL positive staining in the HRT, all belonging to the HVL-MEC group. TUNEL staining foci were moderately to widely distributed in fetal THY and LVR and less abundant in the fetal HRT (Fig. 1). The limited TUNEL staining in the control group was within normal expectations (Additional file 1).

**Fig. 2:**
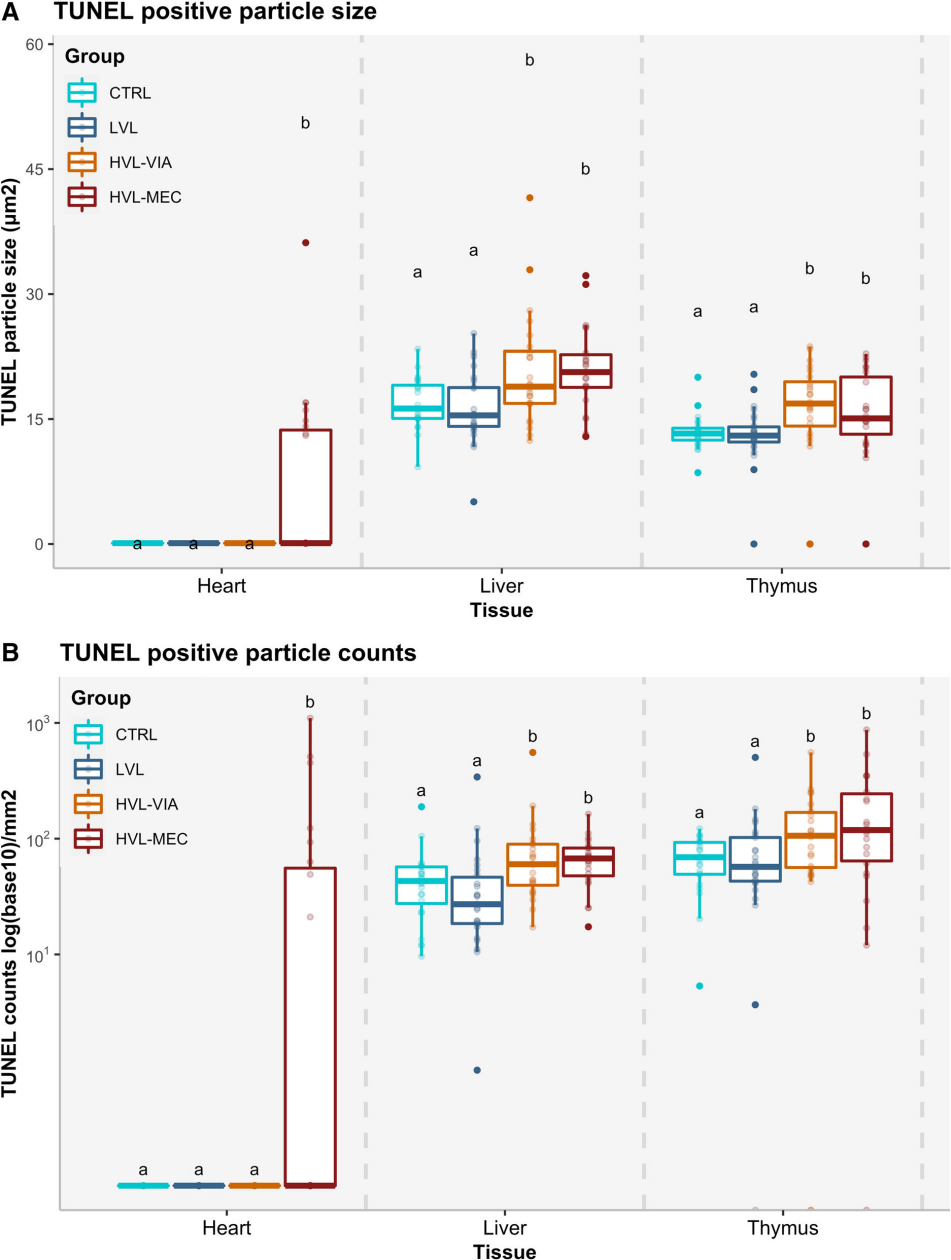
Box-and-whisker plot of TUNEL positive foci counts and sizes by group. The Y-axis presents the size or counts, the X-axis presents the tissues analyzed. Each group is represented by a different colour. **A** Size of TUNEL positive foci/μm^2^ in fetal heart, liver and thymus and **B** counts/mm^2^ of TUNEL positive staining

In the liver, TUNEL positive staining was distributed in all areas and found in hepatocyte nuclei (Fig. 1). The mean size of TUNEL positive foci (Fig. 2a) in the liver was significantly (*P* = 0.0009) larger in the HVL-VIA and HVL-MEC groups (20 ± 6.7 μm^2^ and 21 ± 5 μm^2^, respectively) compared to the CTRL and LVL groups (17 ± 3 μm^2^ and 16 ± 4.2 μm^2^, respectively). The number of TUNEL positive foci (Fig. 2b) was significantly (*P* = 0.0004) greater in HVL-VIA and HVL-MEC fetuses (average 86.9 ± 107.4 and 68.3 ± 32, respectively) compared to the CTRL (average 48.9 ± 36.8) and LVL (average 46.5 ± 66.1) groups.

In thymus, TUNEL positive cells were diffusely distributed along the cortical and cortico-medullar regions, located in the thymocytes, mostly as single cell staining and rarely presenting in clusters. The number of stained foci differed among groups (*P* = 0.009) with the CTRL and LVL groups having similar counts; 66.2 ± 29.6 and 87.5 ± 95.7 positive stained foci on average, compared to 131.4 ± 118.7 and 192.5 ± 202.3 positive stained foci for the HVL-VIA and HVL-MEC groups, respectively. Furthermore, the mean size of TUNEL positive foci in thymus was significantly (*P* = 0.0002) larger in the highly infected animals (HVL-VIA = 16.8 ± 5.9 μm^2^ and HVL-MEC = 16.3 ± 5.1 μm^2^) compared to the CTRL and LVL-VIA animals (13.5 ± 2.1 μm^2^and 13.3 ± 3.6 μm^2^, respectively).

Only eight of the 100 fetal hearts, all from HVL-MEC fetuses, had any positive TUNEL staining and in these fetuses the staining was located in the cardiomyocytes. The mean number of TUNEL positive foci were 301.5 ± 373.9 in HVL-MEC fetuses. The average size of positive stained foci was 17.6 ± 7.6 μm^2^ for HVL-MEC fetuses.

The TUNEL staining foci size on thymus tissues was significantly correlated (*P* < 0.05) to vasculitis distribution and severity in MFI, as well as endometrial inflammation, previously analysed for these animals [13]. Similarly, the foci counts on fetal liver were also correlated to vasculitis severity in MFI. Although the correlations were not strong (Table 1), it indicates a progression in or aggravation of in fetal disease concomitant with progression of lesion severity on the MFI.

**Table 1:** Spearman’s rank correlation test results between liver or thymus (TUNEL foci counts or sizes) and maternal-fetal interface (MFI) lesion severity^a^

	LIVER		THYMUS	
	SIZE Rho/***P***-value	COUNTS Rho/***P***-value	SIZE Rho/***P***-value	COUNTS Rho/***P***-value
**Vasculitis distribution**	0.16 / 0.11	0.19 / 0.06	**0.21 / 0.04**	0.14 / 0.18
**Vasculitis severity**	0.18 / 0.07	**0.24 / 0.01**	**0.24 / 0.02**	0.17 / 0.10
**Endometrial inflammation**	0.16 / 0.11	0.13 / 0.18	**0.23 / 0.02**	0.15 / 0.15
**Myometrial inflammation**	0.01 / 0.90	−0.00 / 0.98	0.05 / 0.59	−0.09 / 0.38

^a^MFI lesion scores as previously evaluated [13]

### Experiment 2: apoptosis and hypoxia gene expression in fetal tissues at 12 dpi

**Fig. 3:**
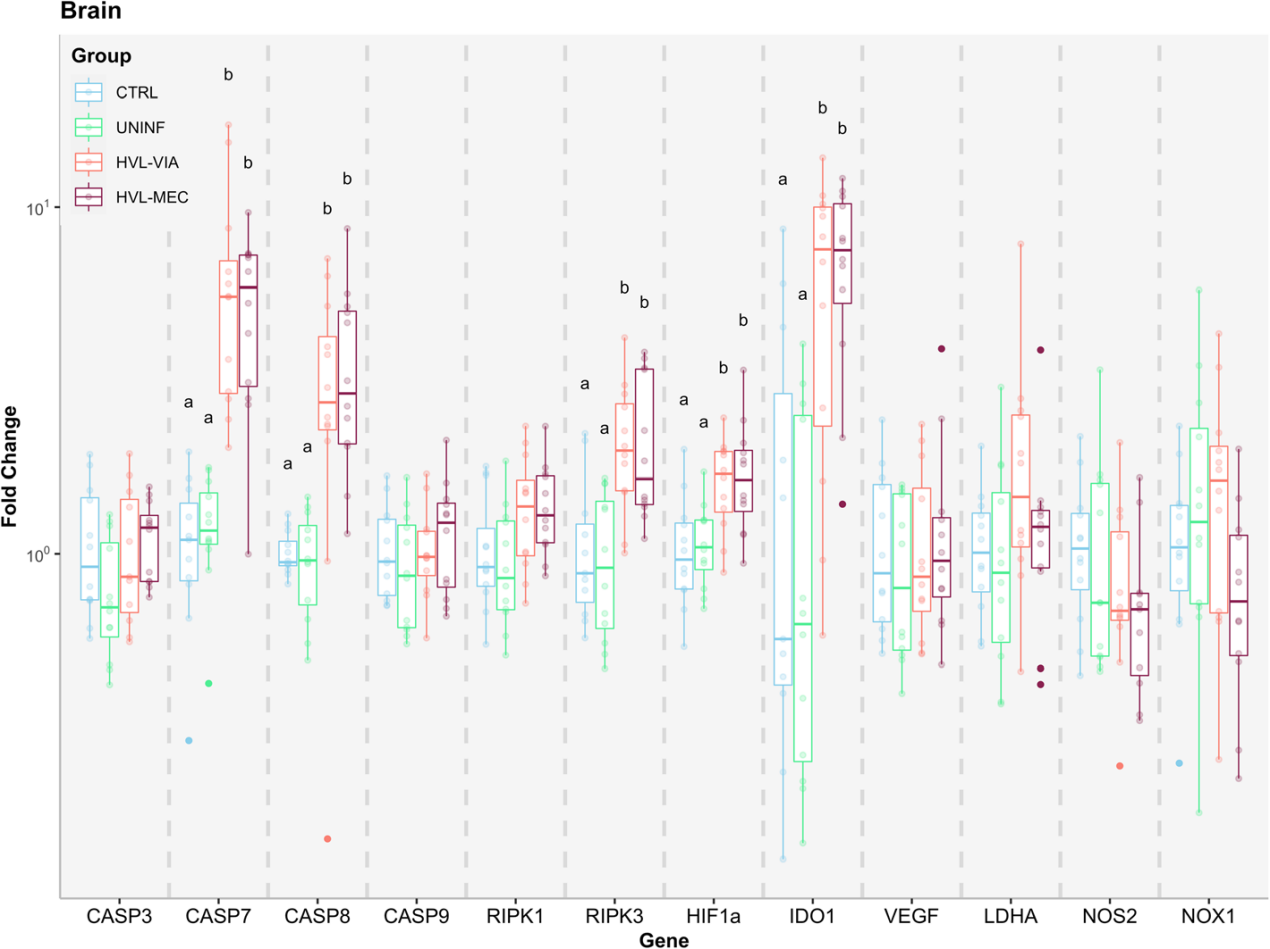
Apoptosis and hypoxia gene expression in fetal brain. Box-and-whisker plot of twelve target genes analyzed in fetal brain for each of the four fetal phenotypic groups (represented by different colours). The Y-axis presents fold changes, the X-axis presents the targeted genes. Superscript letters indicate statistical differences among groups for individual genes (*P* < 0.05)

A number of genes were significantly upregulated following fetal infection (*P* < 0.05). The expression of IDO1 was significantly increased in the BRN of HVL-VIA and HVL-MEC fetuses, consistent with PRRSV infection (Fig. 3). HIF-1α was also significantly elevated in BRN of both PRRSV-infected groups, possibly indicating the activation of hypoxia mechanisms. Additionally, significant upregulation of CASP7, CASP8 and RIPK3 genes of HVL-VIA and HVL-MEC groups indicate the activation of the apoptosis pathway in fetal BRN. The expression of no other genes differed significantly from CTRL.

**Fig. 4:**
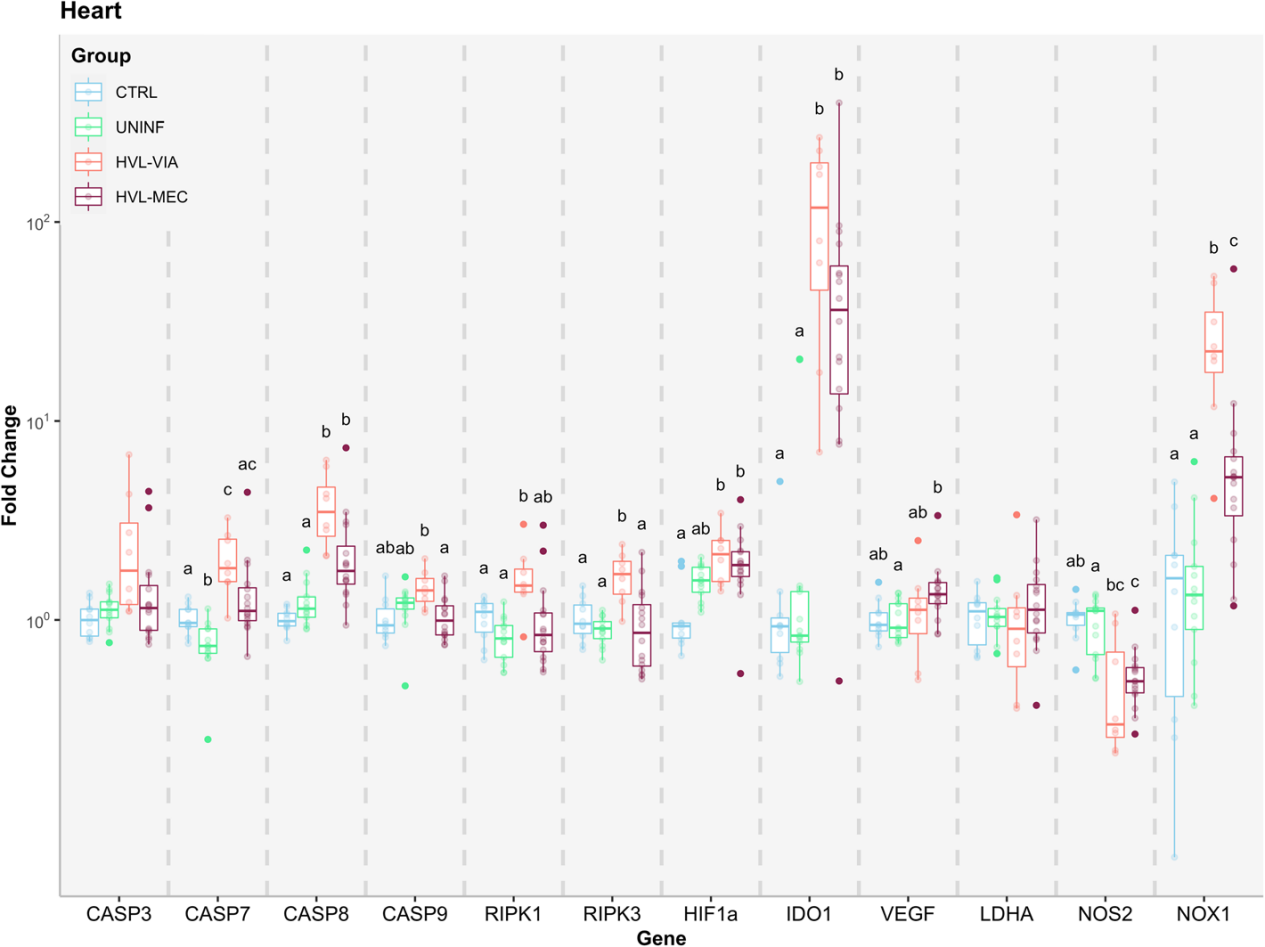
Apoptosis and hypoxia gene expression in fetal heart. Box-and-whisker plot of twelve target genes analyzed in fetal heart for each of the four fetal phenotypic groups (represented by different colours). The Y-axis presents the fold change, the X-axis presents the targeted genes. Superscript letters indicate statistical differences among groups for individual genes (*P* < 0.05)

Of the three tissues examined, fetal heart was the most affected (Fig. 4). The expression of all tested genes except for CASP3 and LDHA were significantly (*P* < 0.05) altered compared to CTRL. The marked upregulation of IDO1 (almost 10^2^ fold change) in HVL-VIA and HVL-MEC fetuses is indicative of the infection of the HRT. The HVL-VIA group had the most significant upregulation of genes related to apoptosis (CASP7, CASP8, RIPK1, and RIPK3), whereas only CASP8 was significantly upregulated in HVL-MEC compared to both non-infected groups. CASP9 gene expression did not differ among HVL-VIA versus CTRL and UNINF groups but was significantly upregulated compared to HVL-MEC. With the exception of CASP8, HVL-MEC did not differ significantly from CTRL and also did not differ significantly from HVL-VIA in the expression of CASP7, CASP8 and RIPK1. The UNINF group had expression patterns similar to the CTRL group in most of the targeted genes but presented significant downregulation of CASP7. With regards to the hypoxia related genes, HIF-1α was significantly upregulated in both HVL-VIA and HVL-MEC, while the UNINF group was intermediary between CTRL and the infected groups. The HVL-VIA group showed marked upregulated expression of NOX1, while HVL-MEC were intermediary between HVL-VIA and both non-infected groups. The only downregulated gene in the infected animals was NOS2, where HVL-MEC was significantly different from the CTRL group and HVL-VIA was intermediary between CTRL and HVL-MEC. The UNINF group was similar to the CTRL group in all genes related to hypoxia.

**Fig. 5:**
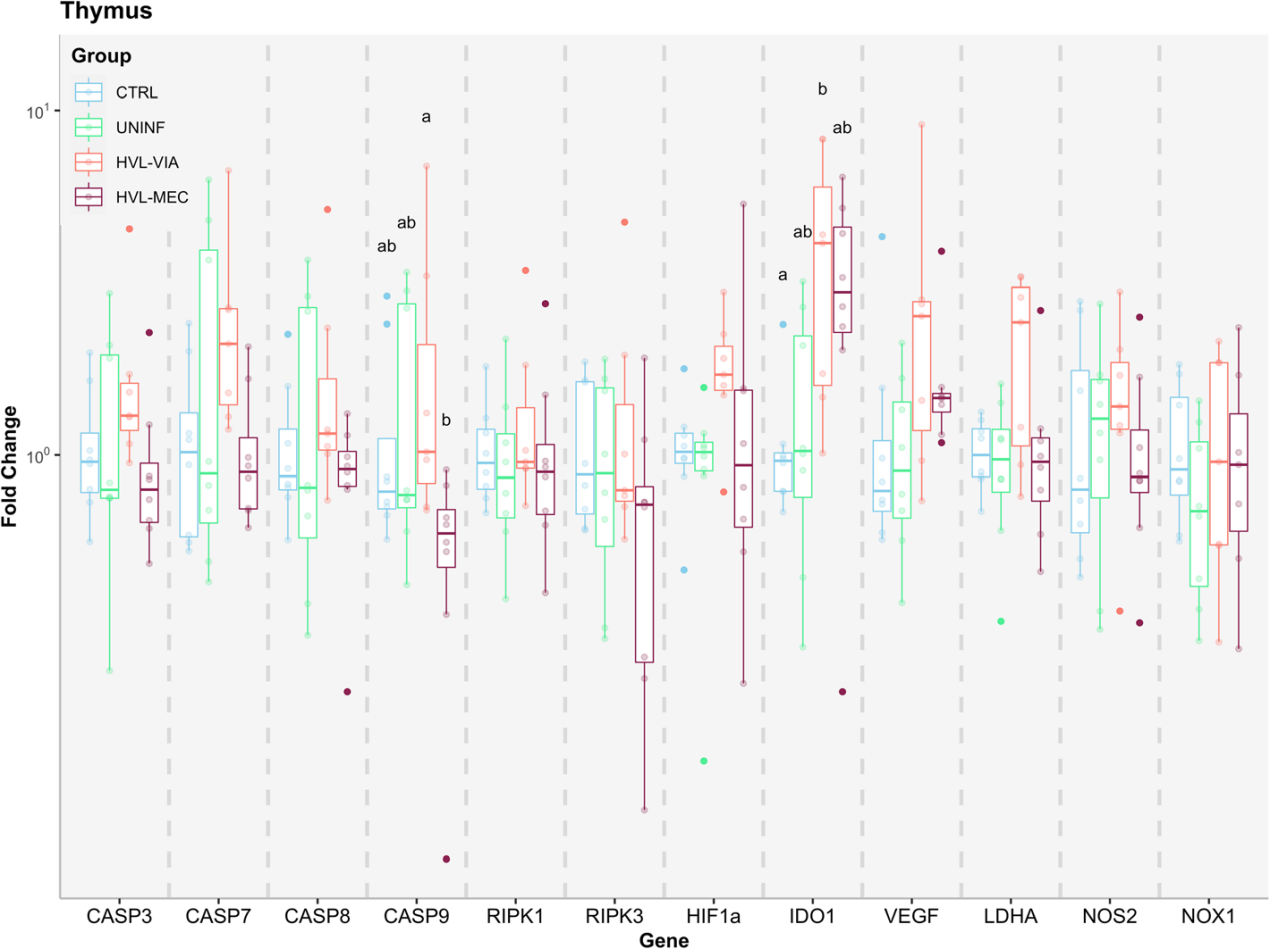
Apoptosis and hypoxia gene expression in fetal thymus. Box-and-whisker plot of twelve target genes analyzed in fetal thymus for each of the four fetal phenotypic groups (represented by different colours). The Y-axis presents the fold change, the X-axis presents the targeted genes. Superscript letters indicate statistical differences among groups for individual genes (*P* < 0.05)

Fetal thymus was the least affected tissue in this study (Fig. 5). The HVL-VIA group had a significant upregulation of IDO1, consistent with PRRSV-infection. Expression of IDO1 was numerically increased in HVL-MEC except for one fetus in this group. There is no explanation for this outlier fetus (G189-R3), as the fetus was phenotypically representative of the group. Three UNINF and one CTRL fetus also had upregulated IDO1 expression despite being uninfected. The only other altered gene among groups was CASP9, which was significantly downregulated in HVL-MEC compared to the HVL-VIA, but neither of these groups differed from the CTRL and UNINF groups. No correlation was found between target genes fold change and vasculitis in endometrium or placenta, endometritis, placentitis, and placental detachment (unpublished data).

**Fig. 6:**
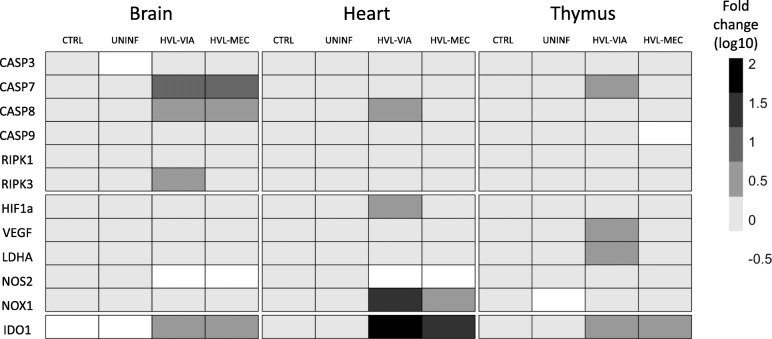
Summary of gene expression fold changes by fetal tissues and group. Heat map of group median gene expression fold changes associated with apoptosis (CASP 3, 7, 8, 9; RIPK 1, 3) and hypoxia (HIF1a, VEGF, LDHA, NOS2, NOX1) by tissue and fetal phenotype. IDO1 gene expression is an indicator of PRRSV infection. Higher fold changes are represented by more intense colours (grey scale white to black)

A heatmap summarizing the gene expression findings is presented in Fig. 6.

## Discussion

Although first described over 30 years ago in North America, Europe and Asia (as reviewed by Christianson [20]), porcine reproductive and respiratory syndrome (PRRS) is still one of the most damaging diseases affecting the pork industry. The reproductive form of the disease is responsible for 45% of all economic losses caused by PRRS virus infection [1]. The infection of pregnant gilts and sows in their last third of gestation often results in only mild clinical signs in the dam, however, the litter can be severely affected by abortions, fetal death, weak born piglets and pre-weaning mortality [21]. The mechanisms of maternal uterine infection and transplacental transmission have been explored [8–10, 13, 22, 23] and a few hypothesis about transplacental infection have been proposed and are reviewed in detail elsewhere [24]. In spite of the rapid development of severe endometritis, placentitis, and endometrial vasculitis subsequent to PRRSV infection, it is uncertain if those lesions result in fetal death, since fetuses present with minimal and non-lethal lesions when found dead [4, 14]. However, previous studies have concluded that there is no relationship between MFI lesions or viral load and fetal death [7, 12]. Thus, our goal was to investigate the fetal mechanisms that are potentially leading to compromise and death following PRRSV infection of third trimester fetuses.

Two separate animal experiments were conducted aiming to find possible insights into fetal death mechanisms following PRRSV infection. Our first experiment investigated apoptosis in fetal tissues after 21 days of maternal inoculation, using paraffin embedded samples from a previously described project [25] conducted in 2012. This was a follow up to our previous investigation in which apoptosis in the MFI (determined by TUNEL staining) was positively associated with PRRSV concentration in the fetal thymus and to meconium-staining of the fetus [13]. The results from this first experiment guided our second experiment that was designed to investigate an earlier stage of apoptosis at 12 days post-infection (12 DPI) through gene expression. The snap frozen tissues for this experiment were collected from a 2017 animal experiment. We also investigated the role of hypoxia as previous research suggested its involvement in fetal compromise [7, 14]. Unfortunately, not all “Experiment 2” fetal samples collected yielded high quality mRNA, explaining the difference in tissue samples used between experiments.

The four fetal phenotypic groups selected for these experiments are representative of the evolution of PRRSV fetal infection and disease. The UNINF and LVL-VIA groups contained either non-infected or very low viral load fetuses from inoculated/infected dams. These are considered the most resistant fetuses [18], escaping infection entirely or sustaining a low rate of viral replication resulting in low levels of virus detectable in SER, THY, and PLC. UNINF fetuses are rather rare, although they would be of high value to the pork industry. Our use of the four fetal phenotypic groups also enabled investigation of the mechanisms that may contribute to fetal death and whether those mechanisms are initiative by maternal or fetal infection; i.e., if they are initiated only after the fetuses per se becomes infected or if there is a threshold of viral concentration to be achieved before disease starts.

Although it is unknow if UNINF fetuses would have been alive and healthy if gestation was taken to term (gestation day 115), they were more resistant than the HVL-VIA and HVL-MEC fetuses at the termination of the experiment (gestation day 106). By contrast, the HVL-VIA fetuses were the most tolerant or resilient fetuses [18] because in spite of high viral load, they remain viable. The HVL-MEC group was the most susceptible [18]. Fetal meconium-staining is largely recognized as a sign of multi-factorial in-utero distress that leads the fetus to defecate or regurgitate into the amniotic sac resulting in contamination of the skin (and sometimes lung) by this yellowish fecal matter. A positive relationship between high PRRSV viral load and meconium-staining of the fetus has been previously reported, indicating this is an initial step towards fetal compromise and death after infection [25]. The most susceptible fetuses are those that die following maternal PRRSV infection. Although decomposed and autolyzed fetuses were present in our challenge trials, we did not collect tissue samples from these fetuses because the mRNA quality was subpar.

In our first experiment, TUNEL staining in liver and thymus confirmed the association between DNA fragmentation and HVL-VIA and HVL-MEC fetuses that was previously observed [13], where the number of TUNEL positive foci in the MFI were related to the HVL-VIA and HVL-MEC phenotypes at 21 DPI. The same relationship was found in fetal heart samples with HVL-MEC fetuses having greater counts of TUNEL positive foci than the CTRL, LVL-VIA and HVL-VIA groups. This result also agrees with a recent study investigating disruption of the fetal thyroid hormones in PRRSV-infected fetuses [18], where the HVL-MEC group was more affected than the respective CTRL, UNINF, and HVL-VIA groups. The diffuse distribution of the apoptotic foci in the liver and thymus may indicate the apoptotic process is advanced in those tissues, possibly contributing to fetal demise.

By evaluating the size of TUNEL positive foci we aimed to determine any association with the progression of the disease and/or viral load of fetuses. Although no standards to characterize these findings by size exist to our knowledge, we hypothesize that it can, at least, indicate the abundance and extent of the apoptotic process in the affected tissues. Statistically, there were differences among the groups, where the highly infected animals (HVL-VIA and HVL-MEC in LVR and THY, HVL-MEC in HRT) presented with larger foci than the non-infected groups (UNINF, CTRL). We interpret these results as confirmation that the highly infected animals are in a more severe state of apoptosis, which may contribute to fetal demise or death.

Based on insights from the first experiment using 21 DPI tissues, we investigated an earlier timepoint (12 DPI) to assess gene expression of apoptosis initiators and executioners. Apoptosis is the programmed cell death, regulated by a family of cysteine proteases (caspases; CASP) [15] along two main pathways: intrinsic and extrinsic. The intrinsic pathway is very well-regulated and initiated by mitochondrial perturbation resulting from cellular stress or cytotoxic insults. It is mainly regulated by CASP-9, an initiator caspase, that subsequently activates the effector, CASP-3 [15]. PRRSV activates the extrinsic apoptotic pathway after infecting the MFI [9, 13], which can be initiated via the TNF receptor (TNFR) family coupled to extrinsic signals. The extrinsic pathway is mainly regulated by the initiator, CASP-8, which subsequently activates CASP-3 and CASP-7 [15].

The TNFR and other inflammatory signals can also initiate the necroptosis pathway. Necroptosis is another form of programmed cell death that shares common reactions and processes with both apoptosis and necrosis. Also classified as a “programmed cell necrosis”, it is mainly regulated by the receptor-interacting protein kinases 1 and 3 (RIPK1 and RIPK3) [26]. Although there are many cells and signals involved in the extrinsic apoptotic pathway and the necroptosis pathway, we focused our investigation on expression of the main genes involved in both (to which we will refer here simply as “apoptosis”) in fetal tissues after maternal PRRSV inoculation.

There was a remarkable difference in gene expression across tissues and phenotypic groups. Apoptosis related genes were typically over expressed in brain and heart of PRRSV-infected fetuses, confirming that infection induces cell death in fetal tissues. Importantly, this apoptotic fetal response is dependent on infection of the fetus, not by maternal infection as noted by the lack of statistical difference between the UNINF and CTRL groups. It is also in general agreement with previous findings, where increased counts of apoptotic cells in the MFI were associated with increased fetal thymus PRRSV viral load [13].

One of the most unexpected and potentially important findings is that heart was the most affected organ, presenting with the higher number of altered genes and higher fold change averages among the three analyzed tissues. This aligns with the findings of Pasternak [18], where after fetal PRRSV infection genes related to cell cycle progression were downregulated and genes related to cell cycle inhibition were upregulated in the fetal heart. Moreover, this result partially agrees with our TUNEL findings, where evidence of apoptosis was only found in the hearts of HVL-MEC group, whereas gene expression was more consistently altered in HVL-VIA fetuses. Additionally, early experiments demonstrated the presence, albeit inconsistent, of virus in the heart of PRRSV challenged fetuses using culture recovery methods [27]. This finding is consistent with immune histochemical analysis of the postnatal heart of piglets following in utero infection [28, 29]. The degree of apoptosis and gene expression changes in fetal heart following local infection may therefore be a key indicator of fetal outcome. Interestingly, fetal thymus, the putative primary site of PRRSV replication on the fetus [27], despite presenting with greater counts and larger sizes of TUNEL positive foci in infected fetuses at 21 DPI, was surprisingly the least altered tissue, having only two out of 12 genes differentially expressed among groups of fetuses. This finding may be due to the early timepoint of sample collection, or to the fact that, as the main viral replication site, the immunosuppressing characteristics of the virus [30] are more influential in thymus than other tissues. Although thymus is considered a tissue of great importance in fetal PRRSV infection, it is not a vital organ for fetal survival in the acute stages of the disease and may therefore only be relevant in the experimental context as a measure of fetal infection.

Hypoxia, the deprivation of adequate levels of oxygen in a tissue, has been theorized as a potential cause of PRRSV-related fetal death based on the observation of umbilical cord lesions [14], which were found to be associated with meconium-staining of the fetus [8]. As some umbilical cord lesions have been observed in dead fetuses from PRRSV infected dams, it is plausible that a reduced blood flow would lead to decreased levels of available oxygen for the fetus. Inflammatory lesions within the MFI and placental detachment may also lead to reduced maternal oxygen transfer to the progeny. These theories prompted our interest in testing the main genes related to hypoxia and its consequences. HIF-1α, a member of the hypoxia-inducible factors (HIFs), is a master regulator of the adaptive response to low levels of oxygen [31]. It is ubiquitously expressed and under normal conditions (normoxia), its subunits are hydroxylated by prolyl hydroxylases (PHD1–3) and go under proteasomal degradation by the von Hippel Lindau (VHL) E3 ubiquitin ligase complex [31]. However, during hypoxia the PHD enzymes are unable to function, suppressing HIF-1α hydroxylation and stabilizing its subunits. From there, HIF-1α can dimerize with HIF-1β and promote transcription of other hypoxia-responsive target genes such as vascular endothelial growth factor (VEGFα), lactate dehydrogenase A (LDHA), nitric oxide synthase 2 (NOS2), and NADPH oxidase 1 (NOX1) [31, 32]. In fetal brain, expression of HIF-1α was upregulated in highly infected fetuses (HVL-VIA and HVL-MEC) compared to CTRL and UNINF, however, none of the other hypoxia-responsive genes were altered. This might indicate an early response to hypoxia or no response at all. HIF-1α can be also involved in the cell death cycle by activating expression of genes in the apoptosis pathway [33], which may explain its upregulation. By contrast, the heart of highly infected fetuses presented with multiple altered genes in addition to HIF-1α. The VEGFα gene was slightly increased in HVL-VIA (1.07 fold changes) and HVL-MEC (1.25 fold changes) groups, suggesting an increase in the proliferation and migration of vascular endothelial cells to provide a larger vascular network for oxygen exchange in response to hypoxia. Much greater differences in expression were observed in the NOX1 (NADPH oxidase 1) gene in the heart of infected animals. NOX1 is involved in many physiological pathways, but it is normally responsible for catalyzing the production of reactive oxygen species (ROS) involved in vascular system. ROS are produced in very low amounts under normal situations, however, ROS can be induced by an increase of NOX1 resulting in damage to cellular proteins, RNA, DNA and lipids. More importantly in this scenario, it impairs the PHD enzymes, decreasing the hydroxylation of HIF-1α [34]. In any case the degree of disruption within this pathway is consistent with previous work by Pasternak et al. [18] showing the high degree of susceptibility of this critical organ to PRRSV infection.

The downregulation of inducible nitric oxide synthase gene (iNOS or NOS2) in the HVL-MEC fetuses (and a trend for the HVL-VIA group) was interesting and unexpected. The production of this enzyme can be induced by inflammatory cytokines such as TNF-α and INF-gamma that are present in sera of PRRSV infected fetuses at or over 19 DPI [17, 27]. NOS2 induces dilatation of blood vessels in response to hypoxia in order to increase blood (and oxygen) availability [35]. The unexpected result might indicate the levels of TNF-α and INF-gamma might not be sufficiently increased at the termination point (12 DPI) used in this project to cause the expected effects. As none of the hypoxia related genes were altered in THY, it is possible that this tissue is not experiencing any hypoxia. However, it’s important to note that in mice the thymus was observed to be a naturally hypoxic tissue when compared to other organs and blood cells, enabling the thymus to be better adapted to hypoxia [36]. This may explain why the expected responses was not initiated. Moreover, as T cells have a lower activation in hypoxic environments [37], this might be a factor that makes thymus a preferred tissue for PRRSV replication.

The only gene that presented a constant pattern throughout all the tissues was indoleamine 2, 3-dioxygenase 1 (IDO1), where its expression was consistently upregulated in highly infected fetuses. This enzyme facilitates tryptophan metabolism and the production of kynurenine [38], which interestingly, was increased in HVL-VIA fetuses compared with UNINF fetuses [39]. Kynurenine is involved in the “metabolic immune regulation” mechanism and its gene expression can be regulated by TNF-α and INF-gamma, as reviewed by Richardson [38]. Because expression in the UNINF and CTRL groups were similar, we deduce that this response to infection is only elicited when the virus infects the fetus (not only the dam), confirming a fetal compartmentalized response to PRRSV infection.

## Conclusions

The findings of this study bring new insights into fetal demise mechanisms after maternal inoculation with PRRSV. Apoptosis is clearly a compromising mechanism evident in multiple organs in the highly infected fetuses. It is plausible the observed apoptotic tendencies, particularly within the heart, contribute to fetal compromise and death beginning as early as 12 DPI and still evident at 21 DPI. Both HVL groups (−VIA and -MEC) displayed indications of an apoptotic process in the brain, while fetal heart is the most severely affected fetal organ following PRRSV infection, showing signs of hypoxia in addition to the apoptosis process.

## Material and methods

### Experiment 1: apoptosis in fetal tissue at 21 DPI

#### Animals

Animal work was conducted in strict accordance with the guidelines of the Canadian Council of Animal Care and with approval of the University of Saskatchewan’s Animal Research Ethics Board (Protocol #20110102). This project has been described in detail elsewhere [25] and complied to the ARRIVE Guidelines (https://arriveguidelines.org/arrive-guidelines) where feasible. In short, 133 purebred Landrace gilts bred to York boars were transported, ﻿in bi-weekly repetitions, to a biocontainment level 2 (BCL2) animal facility at University of Saskatchewan at day 80 of gestation. After 5 days of acclimation, 114 randomly selected gilts were inoculated with 1 × 10^5^ TCID_50_ PRRSV NVSL 97–7895; 2 mL intramuscular (IM) and 1 mL into each nostril. Nineteen gilts were mock inoculated with minimal essential medium to serve as control animals.

#### Sample collection

At 21 DPI, gilts were humanly euthanized ﻿by intravenous barbiturate overdose followed by cranial captive bolt and exsanguination. The uterus was removed, placed linearly and opened enabling fetal assessment. Fetuses were identified and numbered in accordance with their position in the right or left uterine horn. Their preservation status was determined as: viable (VIA live, normal skin color), meconium-stained (MEC; live, normal skin covered with meconium), decomposed (DEC; dead, pale skin, sometimes edematous), autolyzed (AUT; dead, pale or dark skin, friable or liquefied organs) or mummified (MUM; dehydrated, dark brown color, crown-rump length < 20 cm). Samples were not collected from AUT and MUM fetuses. From all other fetuses, liver (LVR), heart apex (HRT), and thymus (THY) were collected into 10% buffered formalin for routine histopathologic processing. Serum (SER) and THY were collected and frozen at − 80 °C for PRRSV RNA quantification.

#### PRRS virus quantification and group selection

PRRSV was quantified in fetal THY and SER by RT-qPCR as previously described [25]. Briefly, ﻿RNA was extracted from 140 μL SER using the QIAamp Viral RNA mini kit (Qiagen Inc., Toronto, ON) or from 10 to 20 mg THY using the RNeasy extraction kit (Qiagen Inc.) according to the manufacturer’s instructions. Primers targeting the ﻿C-terminal end of ORF7 of NVSL 97–7895 were designed and a *Hind*III linearized plasmid, pCR2.1TOPO-NVSL, containing a 446 bp sequence of ORF7 serial dilution was used as a standard curve. The ﻿Master mix (Brilliant II RT-qPCR Low ROX 1-Step Master Mix, Agilent technologies Canada Inc., Mississauga, ON) was used and the reactions were run on a Stratagene MX3500P (Agilent Technologies, Mississauga, ON). Results were transformed to log (base 10).

PRRSV-infected viable fetuses were further divided into a low viral load viable group (LVL-VIA, *n* = 25) with viral load (VL) lower than 4.1 log_10_ copies/μL or mg in both SER and THY (19/25 or 76% had less than 1 log), and a high VL viable group (HVL-VIA, *n* = 25) group with viral load (VL) higher than 5.0 log_10_ copies/μL or mg. In addition, a HVL-MEC group (*n* = 25) was selected to represent the most PRRSV-susceptible set of fetuses. Viral load by group is graphically displayed in Additional file 2. In addition to meeting viral load criteria, nearly all fetuses had been included in a previous study [13] investigating apoptosis at the maternal fetal interface using the TUNEL assay. As much as possible, infected fetuses from multiple phenotypic groups were nested within litter. In fulfilment of these criteria, the 75 LVL-VIA, HVL-VIA and HVL-MEC fetuses originated from 48 PRRSV-challenged gilts (23 providing more than 1 fetal group). Control (CTRL) fetuses (*n* = 25) were derived from 6 non-challenged gilts (4–5 per gilt) and all were included in the previous apoptosis study of MFI tissues [13].

#### TUNEL assay of fetal tissues

As previously described [13], formalin-fixed paraffin embedded fetal organ samples were deparaffinized and rehydrated, followed by protein digestion using Proteinase K solution (15 min). Hydrogen peroxide (3%) was applied (5 min) to block endogenous peroxidase activity, followed immediately by application of the equilibration buffer provided with the ApopTag Plus Peroxidase In Situ Apoptosis Detection Kit (Millipore Canada Ltd., Etobicoke, Ontario) for 10 s at room temperature. Terminal deoxynucleotidyl transferase (TdT) enzyme was then applied at 37 °C for 1 h in a humidity chamber. Slides were rinsed and the signal was revealed using 3-Amino-9-Ethylcarbazole (AEC) chromogen for 15 min. The whole slides were scanned at 20X magnification using OlyVIA (Olympus Corp., Tokyo, Japan), and converted to JPEG format. Image conversion was conducted using ImageJ (version 1.50i) and the quantitative analysis of TUNEL positive staining was completed using ﻿ImagePro Premier software (Media Cybernetics, Inc., Rockville, MD, USA) using the parent-child application for ten random microscopic fields for heart, brain and thymus.

#### Statistical analysis

The raw count data was exported to Microsoft Excel and Stata (StataCorp, College Station, TX) to categorize TUNEL positive staining counts by foci size and to calculate the average foci size per μm^2^ of tissue for each size category. Three size categories were established: “large foci” above 23 μm^2^; “medium foci” between 5 and 23 μm^2^; and “small foci” below 5 μm^2^. The “small foci” category was removed from further analyses because they were considered artifacts, or not related to cell-associated PRRSV infection, as PRRSV infection is restricted to porcine alveolar macrophages or differentiated blood monocytes [40], which measure between 15 and 20 μm [5]. Data was tested for normality using the Shapiro-Wilk test. Differences among the fetal groups in the number of positive TUNEL positive foci counts for each tissue were examined using Kruskal-Wallis and post hoc Dunn’s tests and a Benjamini-Hochberg multiple comparison adjustment. The strength of relationship between fold changes of each target gene and MFI lesions was assessed using Spearman’s rank correlation test, with significance *P* < 0.05, and correlation as: none (rho = 0), weak (0.1 < | rho | < 0.3), moderate (0.3 < | rho | < 0.6), strong (0.6 < | rho | < 0.9), and perfect (| rho | > 0.9) for both positive and negative correlations [41]. The pathologic evaluation and scoring of the MFI samples was previously published by Novakovic [13].

### Experiment 2: apoptosis and hypoxia gene expression in fetal tissues at 12 dpi

#### Animals

Animal work was conducted in strict accordance with the guidelines of the Canadian Council of Animal Care and with approval of the University of Saskatchewan’s Animal Research Ethics Board (Protocol #20160023). This project has been described elsewhere [18]. Briefly, 36 purebred Landrace gilts bred to York boars were purchased at 80 days of gestation from the same high health, PRRSV-free farm as used in Experiment 1. For each of 6 weeks, six gilts were transferred to a BCL2 animal facility at the University of Saskatchewan. After 5 days of acclimation, five gilts blocked by sire were inoculated (INOC) as previously described for “Experiment 1”. One gilt housed in a separate room was similarly mock inoculated with minimum essential medium (CTRL). In total, 30 gilts were INOC and six gilts served as CTRL.

#### Sample collection

After 12 DPI, gilts were euthanized and necropsied as described for Experiment 1, as was fetal preservation classification and sample collection. In addition, placenta (PLC) was manually separated from the endometrium (END) and along with fetal brain (BRN) were snap frozen in liquid nitrogen.

#### PRRS virus quantification and group selection

Virus concentration was measured in PLC, SER and THY for each fetus, as previously described [6] and followed the same viral extraction procedures as Experiment 1. Thereafter, a one-step reverse transcriptase qPCR kit (iTaq Universal Probes 1-Step Kit, BioRad, Mississauga, Canada) was employed for the absolute quantification of viral RNA, using the same primers [25], standard curve, and quality control samples as in Experiment 1. Based on the quantification results, the fetuses were further classified as uninfected (UNINF) representing the fetuses from inoculated gilts that had no detectable infection, and as high viral load (HVL) with viral load over 4.5 log_10_ target RNA copies per mg or μL in PLC, SER, and THY.

Tissues including brain (BRN, *n* = 12/group, total = 48), HRT (*n* = 8–16/group, total = 47) and THY (*n* = 7–8/group, total = 31) were selected from fetuses of four phenotypic groups based on the preservation status of the fetus and its viral load in PLC, SER, and THY: control fetuses from non-inoculated gilts (CTRL, *n* = 30); viable fetuses from inoculated gilts that escaped infection (UNINF, *n* = 33); viable high viral load fetuses (HVL-VIA, *n* = 24); and high viral load meconium-stained fetuses (HVL-MEC, *n* = 36) (Additional file 3). The number of fetuses per group differed slightly because final selection of samples for analyses was dependent on mRNA integrity, as explained below.

#### mRNA extraction and cDNA library generation

Under liquid nitrogen, the selected fetal tissues were individually ground and RNA extracted using a double precipitation method employing Trizol (Thermofisher Scientific, Carlsbad, Canada), followed by DNase (Thermofisher Scientific, Carlsbad, Canada) treatment. RNA purity was determined using nanodrop spectrophotometry (Thermofisher Scientific, Carlsbad, Canada) and a denaturing agarose gel electrophoresis was used to determine mRNA integrity [42]. Only high-quality samples with discrete 18S and 28S rRNA bands were further analyzed. A cDNA library was created using 2 μg of RNA following instructions for the High-Capacity cDNA Reverse Transcription kit (Thermofisher Scientific, Carlsbad, Canada).

#### RT-qPCR

Genes of interest were selected based on their involvement in response to infection (IDO1), hypoxia (HIF-1α, VEGF α, LDHA, NOS2, and NOX1) and apoptosis (CASP3, CASP7, CASP8, CASP9, RIPK1, and RIPK3). The primers were designed based on the *Sus scrofa* 11.1 genome assembly to match all transcript variants of the RefSeq mRNA sequences and positioned to span exon-exon junctions, when possible (Table 1). Eleven housekeeping genes (RPL19, HPRT1, GAPDH, ACTB, HMBS, YWHAZ, IPO8, STX5, SDHA, PPIA, and TBP) previously designed [17] were tested in each tissue and the most stable ones were used for further analyses, as follow: brain – HPRT, IPO8, YWHAZ; heart – RPL19, SDHA, STX5; and thymus - RPL19, SDHA, STX5. All primers were tested and approved with efficiency over 90% and presenting a single peak melting curve. Samples were run in duplicate, each containing 20 ng of cDNA, by real time PCR using the SsoFast EvaGreen Supermix (BioRad) and CFX qPCR system (BioRad), 95 °C for 2 min followed by 40 cycles of 95 °C for 10 s and individual primer set melt temperature (Table 2) for 45 s.

**Table 2:** Porcine specific primer sequences used to assess fetal apoptosis and hypoxia

Pathway	Target	NCBI Gene ID	Forward primer	Reverse primer	Tm (°C)	Length (bp)
Apoptosis	CASP3	397,244	5′-GTGGGATTGAGACGGACAGT-3′	5′-TTCGCCAGGAATAGTAACCAG-3’	60	114
	CASP7	100,156,777	5′-TCGGTGCAAGACCCTTTTAG-3’	5′-GCCTGGAACTGTGGAATAGG-3’	60	178
	CASP8	595,105	5′-GGAACTGCTTTTCCGAATGA-3’	5′-AGCATGACCCTGTAGGCAGA-3’	60	126
	CASP9	100,518,913	5′-TCTGCCCACACCTAGTGACA-3’	5′-ACAGCATTGGAGACCCTGAG-3’	60	171
	RIPK1	100,524,751	5′-ATCCTGTACGGCAACTCTGG-3’	5′-GGTGGTGTTCTCGAAGATGG-3’	60	147
	RIPK3	100,153,263	5′-AATAGGCCCTCCTTCCAAGA-3’	5′-CTCACGGACAGACAACAAGC-3’	60	162
Infection	IDO1	100,519,877	5′-GCTGTCAGAGGGTCTGCTCT-3’	5′-TGAAGGAACTCCACCCACAG-3’	60	139
Hypoxia	HIF1α	396,696	5′-GGCAGCAATGACACAGAAAC-3’	5′-CTGATTGAGTGCAGGGTCAG-3’	59	180
	VEGFα	397,157	5′-CAACATCGCCATGCAGATTA-3’	5′-GCATTCACATTTGTTGTGCTG-3’	60	91
	NOX1	100,739,822	5′-TCCCTTTACCCTGACCTCTG-3’	5′-TCCACCTCAATCCTTGGAAC-3’	60	132
	NOS2	396,859	5′-CTGTGAGACGTTCGATGTCC-3’	5′-GCTGCTGAGAGCTTTGTTGA-3’	59	139
	LDHA	407,245	5′-TTCACCCCCTAAGCTGTCAT-3’	5′-TAAGCACTGTCCACCACCTG-3’	60	178

#### Statistical analyses

The geometric mean of the most stable housekeeping genes in each tissue was used to normalize the expression data. Fold change of each gene of interest was calculated using the 2^-ΔΔCt^ method and univariate non-parametric analysis (Kruskal Wallis followed by pairwise Wilcoxon rank sum test and Bonferroni correction) was performed to determine group differences within genes. All statistical analyses were performed in R [43], data visualization was conducted with the ggplot2 [44] package and observed statistical differences (*P* < 0.05) marked by unique superscripts. The strength of relationship between target genes fold changes and the MFI lesions was assessed using Spearman’s rank correlation test as described above for “Experiment 1” using MFI lesion scores (unpublished data) based on the same scoring previously described [13].

## Supplementary Information

**Additional file 1.** TUNEL staining in three fetal tissues of uninfected control fetuses. Scant cells with TUNEL positive staining are observed at higher magnification of fetal thymus (B) and liver (D), but not in heart (F).

**Additional file 2.** Experiment 1 (21 DPI) PRRSV RNA concentration by fetal group. Viral load expressed in log (base 10) in fetal serum (left) and thymus (right) for each of the four phenotypical fetal groups: non-infected control (CTRL), PRRSV-infected low viral load viable (LVL), PRRSV-infected high viral load viable (HVL-VIA), PRRSV-infected high viral load meconium stained (VHL-MEC).

**Additional file 3.** Experiment 2 (12 DPI) PRRSV RNA concentration by fetal group. Viral load expressed in log (base 10) in fetal serum (left) and thymus (right) for each of the four phenotypical fetal groups in heart (A), brain (B), and thymus (C): non-infected control (CTRL), uninfected fetuses from inoculated dams (UNINF), PRRSV-infected high viral load viable (HVL-VIA), PRRSV-infected high viral load meconium stained (VHL-MEC) fetuses.

## Data Availability

The datasets analysed for Experiments 1 and 2 are available from the corresponding author on reasonable request.

## Abbreviations

PRRSV2: Porcine reproductive and respiratory syndrome virus 2
CTRL: Fetuses from control (non-inoculated) gilts
LVL: Low viral load fetuses (Experiment 1)
UNINF: Uninfected fetuses from inoculated gilts (Experiment 2)
HVL-VIA: Viable high viral load fetuses
HVL-MEC: High viral load meconium-stained fetuses.
DPI: Days post infection
HIF1a: Hypoxia-inducible Factor 1 subunit alpha
IDO1: Indoleamine 2,3-Dioxygenase 1
VEGFa: Vascular endothelial growth factor A
LDHA: Lactate Dehydrogenase A
NOS2: Nitric Oxide Synthase 2
NOX1: NADPH Oxidase 1
CASP3: Caspase 3
CASP7: Caspase 7
CASP8: Caspase 8
CASP9: Caspase 9
RIPK1: Receptor-interacting serine/threonine-protein kinase 1
RIPK3: Receptor-interacting serine/threonine-protein kinase 3
