# Supplementary material for: Fetal hypoxia and apoptosis following maternal porcine reproductive and respiratory syndrome virus (PRRSV) infection
Source: BMC Vet Res. 2021 May 1;17:182. doi: 10.1186/s12917-021-02883-0 (PMC8088663; doi:10.1186/s12917-021-02883-0)

Additional file 1: TUNEL staining in three fetal tissues of uninfected control fetuses. Scant cells with TUNEL positive staining are observed at higher magnification of fetal thymus (B) and liver (D), but not in heart (F).

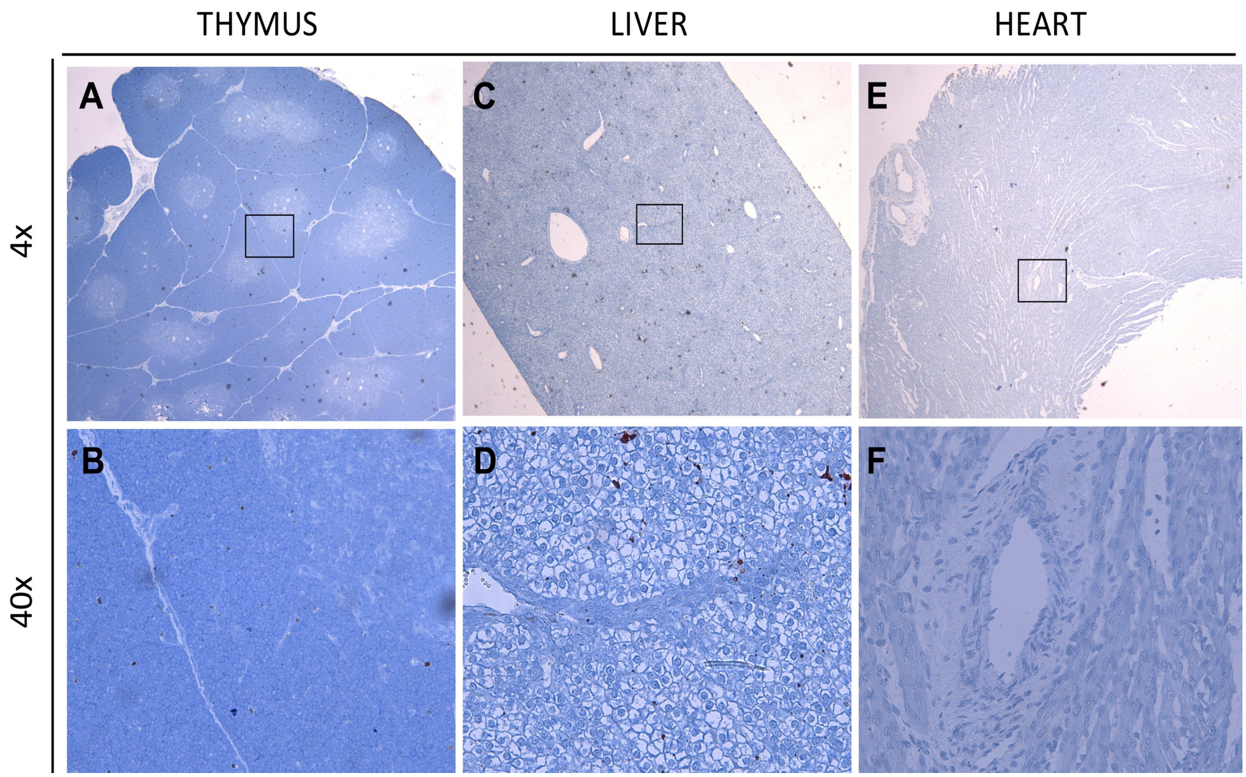

Supplement: Supplementary file 1 — Additional file 1. TUNEL staining in three fetal tissues of uninfected control fetuses. Scant cells with TUNEL positive staining are observed at higher magnification of fetal thymus (B) and liver (D), but not in heart (F). [file 12917_2021_2883_MOESM1_ESM.pdf]
