# Supplementary material for: Fetal hypoxia and apoptosis following maternal porcine reproductive and respiratory syndrome virus (PRRSV) infection
Source: BMC Vet Res. 2021 May 1;17:182. doi: 10.1186/s12917-021-02883-0 (PMC8088663; doi:10.1186/s12917-021-02883-0)

Additional file 2: Experiment 1 (21 DPI) PRRSV RNA concentration by fetal group. Viral load expressed in log (base 10) in fetal serum (left) and thymus (right) for each of the four phenotypical fetal groups: non-infected control (CTRL), PRRSV-infected low viral load viable (LVL), PRRSV-infected high viral load viable (HVL-VIA), PRRSV-infected high viral load meconium stained (VHL-MEC).

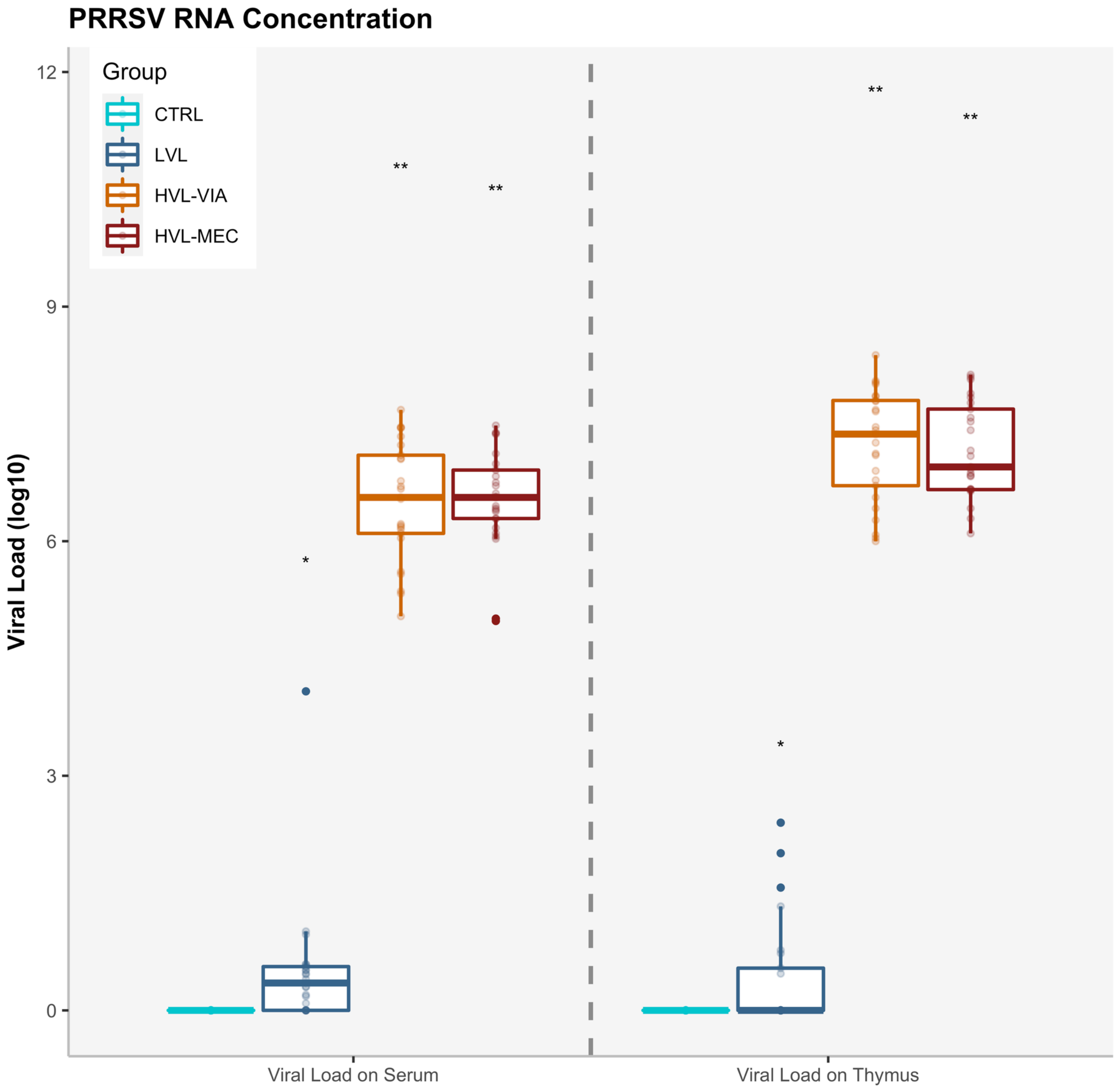

Supplement: Supplementary file 2 — Additional file 2. Experiment 1 (21 DPI) PRRSV RNA concentration by fetal group. Viral load expressed in log (base 10) in fetal serum (left) and thymus (right) for each of the four phenotypical fetal groups: non-infected control (CTRL), PRRSV-infected low viral load viable (LVL), PRRSV-infected high viral load viable (HVL-VIA), PRRSV-infected high viral load meconium stained (VHL-MEC). [file 12917_2021_2883_MOESM2_ESM.pdf]
