# Supplementary material for: Fetal hypoxia and apoptosis following maternal porcine reproductive and respiratory syndrome virus (PRRSV) infection
Source: BMC Vet Res. 2021 May 1;17:182. doi: 10.1186/s12917-021-02883-0 (PMC8088663; doi:10.1186/s12917-021-02883-0)

Additional file 3: Experiment 2 (12 DPI) PRRSV RNA concentration by fetal group. Viral load expressed in log (base 10) in fetal serum (left) and thymus (right) for each of the four phenotypical fetal groups in heart (A), brain (B), and thymus (C): non-infected control (CTRL), uninfected fetuses from inoculated dams (UNINF), PRRSV-infected high viral load viable (HVL-VIA), PRRSV-infected high viral load meconium stained (VHL-MEC) fetuses.

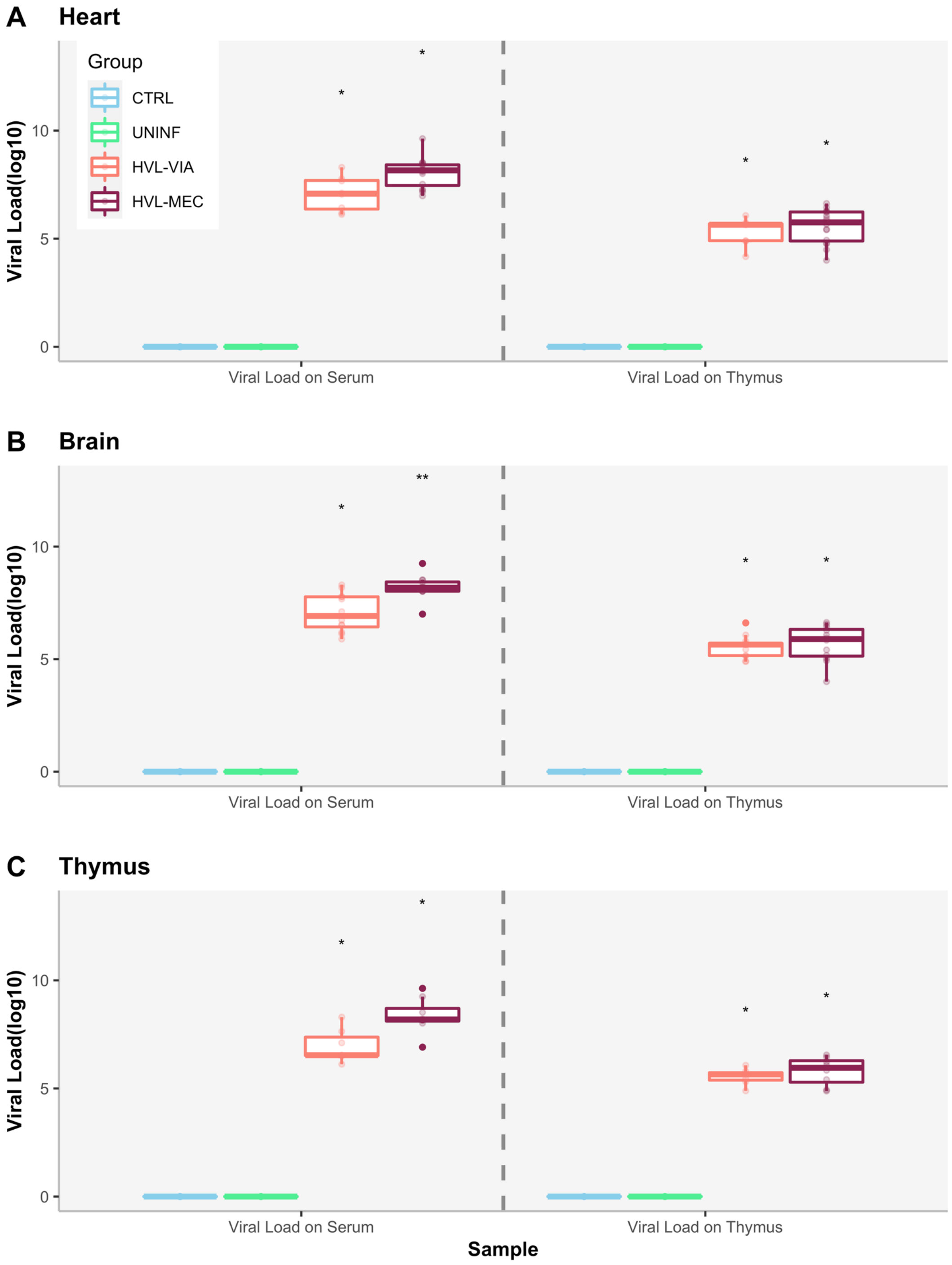

Supplement: Supplementary file 3 — Additional file 3. Experiment 2 (12 DPI) PRRSV RNA concentration by fetal group. Viral load expressed in log (base 10) in fetal serum (left) and thymus (right) for each of the four phenotypical fetal groups in heart (A), brain (B), and thymus (C): non-infected control (CTRL), uninfected fetuses from inoculated dams (UNINF), PRRSV-infected high viral load viable (HVL-VIA), PRRSV-infected high viral load meconium stained (VHL-MEC) fetuses. [file 12917_2021_2883_MOESM3_ESM.pdf]
